# The underlying career values of young adults’ protean and traditional career orientations

**DOI:** 10.1007/s10775-023-09593-z

**Published:** 2023-03-31

**Authors:** Sujin Kim, Michelle Hood, Peter A. Creed, Debra Bath

**Affiliations:** 1 School of Applied Psychology, Griffith University, Parklands Drive, Southport, QLD 4215 Australia; 2 Centre for Work and Organisation Wellbeing, Griffith University, Parklands Drive, Southport, QLD 4215 Australia; 3 Academy, James Cook University, Townsville, Australia

**Keywords:** Protean career, Traditional career, Career values

## Abstract

Although young people espouse a range of career values, the extent to which traditional career values inter-mix with protean values is unclear. We interviewed a group of young university students in Australia (*N* = 24, *M*_Age_ 19.4 years; 50% young men) and examined the full range of traditional and protean values held. Employing applied thematic analysis, we found that freedom/autonomy and fit to self were dominant in protean career themes, while they strongly expressed a desire for job security in a traditional career. The results inform theory development in the career development area and can assist university career counselors.

## Résumé: Les valeurs de carrière sous-jacentes des orientations de carrière protéiformes et traditionnelles des jeunes adultes

Bien que les jeunes adhèrent à un éventail de valeurs professionnelles, la mesure dans laquelle les valeurs professionnelles traditionnelles se mêlent aux valeurs protéiformes n'est pas claire. Nous avons interrogé un groupe de jeunes étudiants universitaires australiens (N = 24, âge moyen 19,4 ans; 50% de jeunes hommes) et examiné l'ensemble des valeurs traditionnelles et protéiformes. En recourant à une analyse thématique appliquée, nous avons constaté que la liberté/l'autonomie et l'adéquation à soi étaient dominantes dans les thèmes de la carrière protéiforme, tandis que les étudiants exprimaient fortement leur désir de sécurité de l'emploi dans une carrière traditionnelle. Les résultats éclairent le développement de la théorie dans le domaine du développement de carrière et peuvent aider les conseillers d'orientation des universités

## Zusammenfassung: Die zugrunde liegenden Karrierewerte der proteanischen und traditionellen Karriereorientierungen junger Erwachsener

Obwohl junge Menschen eine Reihe von beruflichen Werten vertreten, ist unklar, inwieweit sich traditionelle berufliche Werte mit proteischen Werten vermischen. Wir befragten eine Gruppe junger Universitätsstudenten in Australien (N = 24, MAge 19,4 Jahre; 50% junge Männer) und untersuchten das gesamte Spektrum traditioneller und proteanistischer Werte. Mittels angewandter thematischer Analyse fanden wir heraus, dass Freiheit/Autonomie und die Anpassung an das eigene Selbst bei den proteanischen Karrierethemen dominierten, während sie bei einer traditionellen Karriere stark den Wunsch nach Arbeitsplatzsicherheit äußerten. Die Ergebnisse dienen der Theorieentwicklung im Bereich der Karriereentwicklung und können Berufsberatern an Universitäten helfen.

## Resumen: Los valores de carrera subyacentes de las orientaciones profesionales proteicas y tradicionales de los jóvenes adultos

Aunque los jóvenes adoptan una serie de valores profesionales, no está claro hasta qué punto los valores profesionales tradicionales se entremezclan con los valores proteicos. Entrevistamos a un grupo de jóvenes universitarios de Australia (N = 24, MAge 19,4 años; 50% hombres jóvenes) y examinamos toda la gama de valores tradicionales y proteicos. Empleando un análisis temático aplicado, descubrimos que la libertad/autonomía y la adecuación a uno mismo dominaban los temas de la carrera proteica, mientras que expresaban un fuerte deseo de seguridad laboral en una carrera tradicional. Los resultados aportan información para el desarrollo teórico en el área del desarrollo profesional y pueden ayudar a los orientadores profesionales universitarios.

Hall (1996) argued that “the [bureaucratic] career is dead” (p. 8) when highlighting differences between traditional careers, where individuals are loyal and committed to a single organization and “new” career types, which are driven by continuous learning, identity development, and an overarching aim for psychological rather than objective success. Subsequently, many career scholars shifted their focus to examining alternative, new, or contemporary career paths, such as protean, boundaryless, nomadic, and kaleidoscopic careers (Baruch et al., 2015), which are considered parallel or overlapping career types that stand in contrast to the traditional career (Liberato Borges et al., 2015; for review and meta-analysis, see Wiernik & Kostal, 2019). However, the proposition that new career types have superseded traditionally focused career pathways has been contested, with others arguing that traditional career values remain prevalent and are important to consider as they also influence career decision-making, goal setting, and behavior (Clarke, 2013).

Thus, it is important to understand the career values, those self-appraised occupational needs that drive career-related goals, decision-making, and actions (Schein, 1975; Schwartz, 1992), which are held by individuals, especially those held by young people in the earlier stages of career development when they are preparing for entry to the workforce (Monteiro & Almeida, 2015). Career values, which are influenced by a range of individual (e.g., gender) and contextual factors (e.g., socio-economic status), develop early on in childhood, and unfold over the lifespan (Super, 1990). They are learned from parents, peers, and important others, and via education, the media, and cultural norms. Parents, for example, model their work values and reinforce/extinguish those they would like their children to retain/abandon.

A range of new career values have been described for young people (e.g., autonomy, independence; Alonderienė & Šimkevičiūtė, 2018; Creed et al., 2011; Liberato Borges et al., 2015; Rojewski et al., 2017). However, the extent to which these new career values co-exist with more traditional values, such as wanting security and job tenure, remains unclear (Gerber et al., 2009). To address this gap, we employed a qualitative, interview-based research design to identify the range and manifestation of career values expressed by young people. Drawing on a sample of young adults (defined as aged 18–34 years; Australian Bureau of Statistics, 2013) enrolled in professional programs at one Australian university, we contribute to a better understanding of the career values of developing professionals and, thereby, to theory development in this area, while also informing practitioners who assist young people with their careers.

## Protean and traditional career values

Among the new career paths, only protean (Hall, 1976, 1996, 2004) and boundaryless (Arthur & Rousseau, 1996) career concepts have been acknowledged as viable models (Gubler et al., 2014; Herrmann et al., 2015). Theoretically, these are related, yet distinguishable, constructs. The protean approach focuses primarily on internal identity issues, while the boundaryless approach stresses external supports and opportunities across organizations (Briscoe et al., 2006, 2012). However, the boundaryless approach is less applicable for young people with limited work experience as it encompasses physical mobility across business firms (Liberato Borges et al., 2015) and has been criticized for assuming high mobility (Arnold & Cohen, 2008; Rodrigues & Guest, 2010), when individuals still seek structures that give them security and stability (Baruch et al., 2015; Sargent & Domberger, 2007). Thus, our primary focus was on the protean, not the boundaryless, career approach.

A protean career refers to career processes that “the person, not the organization, is managing” (Hall, 1976, p. 201). It can be understood as a collection of career attitudes and values characterized by individual self-directedness (e.g., valuing individual responsibility and accountability rather than relying on career management by the organization) and being values driven (e.g., valuing career success that is meaningful to the individual rather than meeting career objectives valued by the organization; Hall, 2004). Thus, protean individuals manage their career behaviors while being guided by their own career values (Briscoe et al., 2006). Career and life aspirations are self-set and reliant on intrinsic motivation, rather than being driven by dictates from others and external rewards, such as promotion and salary (Hall, 2004; Hall et al., 2018). Protean values also are related to vocational identity, as being clear about one’s career values leads to a more crystallized sense of self, which fosters more self-reliance, confidence, the ability to adapt to changing circumstances, and an acceptance of greater uncertainty and risk (for review, see Hirschi et al., 2017).

Organizational or traditional career values, on the other hand, are characterized by valuing job security, showing long-term loyalty and commitment to one organization, and a desire to succeed by progressing within an organization (Clarke, 2013; Gerber et al., 2009). Organizational career progress occurs through training and mentoring provided largely by the organization, and career success is characterized more by objective rewards and status (Clarke, 2013). In this way, the traditional career path is linear and stepwise, with long-term loyalty and dedication to the organization rewarded with job security and career management (Doden et al., 2018). New career paths, on the other hand, are non-linear, non-hierarchical, multi-organizational, and self-managed (Baruch, 2004). When stable and secure career paths are valued, vocational identity development is a less reflexive and demanding process. The focus is on identifying what job/career opportunities are available, how to make a career decision, and what plans are needed (Super, 1957). As work environments become more uncertain and challenging, however, identity development is no longer a passive process, but requires the individual to reflect on their experiences, needs, and values, and craft a way forward (Law et al., 2002).

It has been argued that the traditional career is fading and will eventually disappear (Baruch et al., 2015). However, this has not received strong empirical support (Clarke, 2013). Gerber et al. (2009) stressed that the traditional career orientation remains prevalent as most employees still prefer long-term employment and job security, and King (2003) found that many early career professionals expect to manage their careers within an organizational structure. Thus, some scholars argue that the dominant dichotomous view of “old” versus “new” career orientation is too simplistic, and a more nuanced understanding of developing career pathways is required (Baruch, 2006; Baruch et al., 2015; Gerber et al., 2009; Sargent & Domberger, 2007; Skromme Granrose & Baccili, 2006). To date, little attention has been paid to the extent to which a perspective that incorporates both traditional and protean orientation elements might co-exist, and we found no studies that specifically examined a mixture of traditional and protean career orientations in young people in the early stages of career development, despite the protean career orientation construct being applied to young people (Alonderienė & Šimkevičiūtė, 2018; Creed et al., 2011; Liberato Borges et al., 2015; Rojewski et al., 2017; Sargent & Domberger, 2007).

## Contemporary career values

Measures devised to assess protean-specific career values (Baruch, 2014; Briscoe et al., 2006; Liberato Borges et al., 2015) assess the two theoretically proposed domains of self-directedness (i.e., autonomy, self-reliance, flexibility, growth, networking, setting own goals, planning ahead, proactivity, and keeping updated) and values directedness (i.e., setting own criteria for success and seeking personal satisfaction, work-life balance, interest, and professional achievement); whereas, scales that measure traditional career values focus on job security, promotion, structured advancement, organizational focus, work-life balance, focus on tenure, loyalty, commitment, and prestige/status (Baruch, 2004; Dawis & Lofquist, 1984; Doden et al., 2018; King, 2003). The value of these specific measures is that they directly assess the protean and traditional values constructs and provide insight into the protean and traditional career orientations.

Some researchers have developed new and revised existing, career values scales to better understand contemporary-held beliefs in adults and young people, rather than relying on traditional conceptualizations and measures (e.g., Dawis & Lofquist, 1984; Schein, 1990; Schwartz, 1992; Super, 1970). Abessolo et al. (2019), for example, devised the Career Values Questionnaire, which assesses eight contemporary career values of social (to help others), management (to supervise others), specialization (to develop expertise), mobility (work that involves travel), independence (set own goals), salary (remuneration), work-life balance (have family-friendly policies), and variety (do interesting work). This scale has the capacity to reflect both traditional (e.g., high scores on management, salary, and status) and protean-like values (e.g., high on independence, low salary concerns), although it does not account for new career values proposed by others, such as being technologically and digitally current (Liberato Borges et al., 2015) and self-actualizing (Sung et al., 2019).

Kuder (2015) revised the Work Values Inventory (Super, 1970), which covers 12 work-related values under three broad headings of work environment (i.e., related to desired/expected supervisory arrangements, work conditions, job security, and level of income), motivation (i.e., desired mental challenge, independence, prestige, and achievement), and type of engagement (i.e., desired variety, lifestyle, creativity, and co-worker relationships). This inventory, which can be used with both adults and young people, also can reflect protean (e.g., high quality lifestyle and independence, and low focus on income) and traditional values (e.g., high-rated job security, prestige, and income focus).

Researchers have proposed that work values have shifted over the past few decades in response to societal and labor market developments, proposing, for example, an increase in importance of intrinsic values (e.g., independence, autonomy, self-realization) and a decline in importance of extrinsic values (e.g., security, status, comfort; Gallie, 2019; Hansen & Leuty, 2012). Such changes, if they exist might be reflected in differences in work values among recent generation cohorts, such as the “silent,” “boomer,” and “X” generations. However, the evidence for this is mixed, and where changes have been identified, they have been small (for reviews, see Hansen & Leuty, 2012; Parry & Urwin, 2011). Changes in work values are likely to be related to cohort, age, and period effects (Parry & Urwin, 2011), and understanding changes in period effects is more likely to shed light on changes resulting from societal influences. Teasing out these overlapping relationships is yet to occur fully, with some studies, for example, showing a decline in the centrality of work across time periods, although these effects are smaller than within-individual changes across their lifetime (i.e., work centrality declines with age; Hajdu & Sik, 2018).

Few studies have set out specifically to examine the mix of protean and traditional career values held today. Focusing primarily on adults, Abessolo et al. (2017a) found that Swiss employees endorsed a mixture of both protean (e.g., autonomy, independence) and more traditional values (e.g., security, status, remuneration). Hauff and Kirchner (2015) tested a large, multi-cultural sample of employees and identified four different groupings based on expressed career values. One group placed importance on job security, high income, and good opportunities for advancement (i.e., high on traditional values), a second group placed less value on security and little value on high income and job opportunities (i.e., a more non-traditional, protean cluster), and the other two were variations on these first two.

In young people, Chow et al. (2017) found that 18-year-olds endorsed both intrinsic (autonomy, interesting work, and sense of achievement, reflecting protean-like values) and extrinsic work values (salary, job security, opportunity for promotion, reflecting more traditional values), although only the intrinsic values were related to later career and life satisfaction. In a qualitative study, Sargent and Domberger (2007) found a mix of protean-like (i.e., prioritizing self-direction, contributing to society, and work-life balance) and traditional-like student groups (i.e., valuing extrinsic achievement, such as promotion and salary, and organizational career management), although there was overlap of values in the two groupings. Last, using cluster analysis, Rojewski et al. (2017) identified distinct protean (e.g., the “protean career architect,” who is driven by personal values and attracted to working across organizational boundaries) and traditional groups (e.g., the “careerist,” who acknowledges career values, is reluctant to take career risks and seeks stability in relationships and organizational settings), although, again, no pure type was identified, rather the different clusters contained different mixes of protean and traditional values.

Some evidence suggests that holding protean career values is advantageous for young people (Alonderienė & Šimkevičiūtė, 2018; Rojewski et al., 2017). For example, Creed et al. (2011) showed that protean values in young adults were related to more self- and environmental exploration and career planning, and Liberato Borges et al. (2015) found that protean values were related to feeling more in control and having better well-being. However, despite this support for new career values, Sortheix et al. (2019) showed that young people strengthened traditional values (e.g., security, tradition, conformity) and weakened more hedonistic ones (e.g., personal growth, self-direction, self-enjoyment) after the 2008 Global Financial Crisis.

There is also support for mixed values. Chow et al. (2017) found that career and life satisfaction at age 45 were related to both intrinsic (e.g., accomplishment, becoming yourself, maintaining interest) and extrinsic values (e.g., security, promotions) measured at age 18, suggesting that both traditional and new career values facilitated better later adjustment. Additionally, Gerber et al. (2009) showed that adult employees, while endorsing career self-management, wanted job security and ongoing tenure, and affirmed organizational commitment, with these values associated with more positive attitudes toward work and the employer. King (2003) found similar results in newly employed graduates who endorsed both new and traditional career values, wanted to develop employability, but aimed to do this with their current employer.

Thus, the evidence suggests that adults and young people express a mix of career values that cover both more traditional hopes and desires (e.g., job security, organizational support for skills development) as well as values related to the protean career (e.g., career self-management, seeking self-satisfaction). Moreover, these value mixes have been shown to be related to improved outcomes for both adults (e.g., improved functioning at work; Chow et al., 2017) and young people (e.g., more successful school-to-work transition; Takeuchi et al., 2021). It is to be expected that traditional career values, which remain prevalent in the community and with parents are still normative for many (Gerber et al., 2009), are now complemented by contemporary career values in response to societal changes (e.g., increased job uncertainty, insecurity, and competition, brought on by wider economic and technological changes; Alonderienė & Šimkevičiūtė, 2018; Sortheix et al., 2019). Indeed, Hall (1996) proposed that people are led inevitably to more of a self-management approach as they are exposed to 21^st^-Century work environments, which reflects an individualism process during which young people adopt values that suit their labor market expectations and have these reinforced, extinguished, or replaced depending on their experiences (Takeuchi et al., 2021).

## Current study

As little is known about the range and mix of career values held by young people today (e.g., how strongly do they retain traditional career values and to what extent have they internalized protean career values), there is a need for additional studies that clarify the underlying values that drive career decision-making and goal pursuit processes in young people. Thus, our primary focus was to describe the extent to which young adults (a) hold protean and traditional values and (b) delineate the manner by which these values are expressed. To achieve the aims, we employed face-to-face interviews and analyzed young tertiary students’ narratives using protean and traditional career value categories. Representative narratives were selected to demonstrate how they express their held values from the perspective of both career orientations. Young tertiary students constitute an appropriate sample as these young people are in the process of appraising their future opportunities, evaluating whether they are suitable for them based on their needs and values, and making decisions regarding career directions (Creed et al., 2009).

## Method

### Participants

Participants were recruited from a large first-year course at one Australian university that taught students from a range of disciplines (e.g., business, public health, law, psychology, and physiotherapy). There were 24 young adults (*M*_Age_ = 19.4 years; *SD* = 2.0; range 17–25; 50% male), all of whom were Australian domestic students, except for one, who was an international student studying in Australia. Most (75%) were working part-time while studying, which is typical for Australian tertiary students (Universities Australia, 2017). See Table 1 for data on individual cases and the presentations of both protean and traditional career values.

**Table 1:** Participant demographic information and presentations of protean and traditional career values; *N* = 24

No	Sex	Age	Background	Type	Degree	Working	Work Type	PCO		TCO
								Self-directedness	Values-driven	
1	F	20	Australian	Domestic	Public Health	Yes	Hospitality	O	O	O
2	M	18	Australian	Domestic	Psychology	No	–	O		O
3	F	17	Australian	Domestic	Physiotherapy	Yes	Hospitality	O	O	O
4	F	18	Australian	Domestic	Psychology	Yes	Retail	O	O	O
5	F	19	Indian	Domestic	Physiotherapy	No	–	O	O	O
6	F	18	Australian	Domestic	Psychology	Yes	Retail	O	O	
7	F	18	Australian	Domestic	Psychology	Yes	Retail	O	O	O
8	M	18	Australian	Domestic	Psychology	Yes	Hospitality	O	O	O
9	F	20	Botswanan	Domestic	Public Health	No	–	O	O	O
10	F	25	Australian	Domestic	Psychology	Yes	Hospitality	O	O	O
11	M	20	Japanese	International	Sports Development	Yes	Hospitality	O	O	O
12	F	19	Australian	Domestic	Physiotherapy	No	–	O	O	O
13	M	18	Sri Lankan	Domestic	Physiotherapy	Yes	Hospitality	O	O	O
14	F	18	Australian	Domestic	Law/Psychology	Yes	Hospitality	O	O	O
15	M	18	Australian	Domestic	Psychology	Yes	Hospitality	O	O	
16	F	21	Australian	Domestic	Psychology	Yes	Hospitality	O	O	O
17	F	23	Australian	Domestic	Psychology	Yes	Hospitality	O	O	O
18	M	23	Australian	Domestic	Psychology	No	–	O	O	O
19	M	18	Australian	Domestic	Psychology	Yes	Video editing	O	O	O
20	M	19	Australian	Domestic	Psychology	Yes	Data entry	O	O	O
21	M	19	Australian	Domestic	Criminology/Psychology	No	–	O	O	O
22	M	22	Australian	Domestic	Physiotherapy	Yes	Sports	O	O	
23	M	19	Australian	Domestic	Physiotherapy	Yes	Retail	O	O	O
24	M	18	Australian	Domestic	Business/Psychology	Yes	Manufacturing	O	O	O

*PCO* protean career orientation, *TCO* traditional career orientation

### Measure

At the start of each interview, participants provided demographic information and verbal consent for the interviews to be audio-recorded for later transcription and analysis. The interview protocol consisted of set introductory questions to orient and relax participants. These were followed by both broad and targeted questions designed to elicit comments about their career values. These questions were interspersed with individualized prompts and encouragements for deeper reflection and elaboration as needed. All questions were designed to elicit participants’ views, beliefs, and values regarding who and what were important in career decision-making, making progress, and succeeding, and what constitutes career success (see Appendix for interview protocol). Interviews, which lasted approximately one hour, were transcribed using the Temi speech recognition software (https://temi.com), which cite 90–95% transcription accuracy. Lastly, the first author corrected any transcription errors within all written transcripts while listening to the whole recordings.

### Procedure

The authors’ university research ethics committee provided ethical clearance for the study. Participants were recruited via their course website where they could register for the study. They were then contacted to arrange a time and place for the interview. In-person, face-to-face interviews were conducted by an independent research assistant, trained, and supervised by the first author. All interviews were conducted in mid-2019 in a quiet space on the university campus, and participants received a $20 shopping voucher as a thank you. As recommended for interview studies (Levitt et al., 2018), interviews were continued until saturation was reached, after which time we suspended recruitment. Saturation was indicated when no new information on our topic was forthcoming.

### Data-analysis

We used a confirmatory thematic analysis approach (Guest et al., 2012) to code the data according to the theoretical career values proposed as protean and traditional (cf. Wiernik & Kostal, 2019), as the main aims of our study were to determine how extensively each set of values was expressed by contemporary young people and to what extent were these values held simultaneously. This approach, which is guided by specific, pre-determined categories, rather than being primarily exploratory, was deemed suitable for the study as it allowed for the exploration of explicitly expressed values of the young participants (Guest et al., 2012). Thus, the themes for the protean values categories were drawn from existing scales and literature (Baruch, 2014; Briscoe et al., 2006; Liberato Borges et al., 2015). There were nine themes for protean self-directedness (freedom/autonomy, self-reliance, flexibility, growth, networking, self-set goals, specific career plans, proactivity, and keeping updated) and six for protean values-driven attitudes (fit to self, suit self, keeping an interest, professional achievement, personal satisfaction, and work-life balance). See Table 2. Drawing on studies by Baruch (2004), Doden et al. (2018), and King (2003), seven themes were identified for traditional values: job security, promotion (structured advancement), organizational focus, focus on tenure, loyalty, commitment, and career success defined as prestige/status. See Table 3.

**Table 2:** Protean career value categories and representative scale items

	Career values	Protean scale items
Self-Directedness	Freedom/autonomy	Freedom and autonomy are driving forces in my career^a^
	Self-reliance	I am in charge of my own career^a^
	Flexibility	For me, career success means having flexibility in my job^a^
	Growth (lifelong learning)	I try to learn the competences needed to improve in a given professional activity^c^
	Networking	I try to establish contacts with professionals who might afford opportunities to develop my career^c^
	Self-set goals	For me, career success is how I am doing against my goals and values^a^
	Specific career plans	I have made career-related plans for the next six months^c^
	Proactivity	I see myself as looking for opportunities to develop my career by myself^c^
	Keeping updated	I am constantly assessing the skills I must acquire to keep myself updated relevant to the job market^c^
Values Driven	Fit to self	I use my own criteria to assess my success in various settings^c^
	Personal satisfaction	In making career decisions, my personal satisfaction comes first^c^
	Work-life balance	I believe that balancing my personal and professional lives is more important than attaining a high career position^c^
	Suit self	The most important factor is my own assessment of my career success, rather than other people’s opinions^c^
	Keeping an interest	If ever I lose interest in the profession I chose, I will look for other occupations that facilitate greater achievements^c^
	Professional achievement	Professional achievement is more important than attaining a high career position^c^

^a^Baruch (2014)

^b^Briscoe et al. (2006)

^c^Liberato Borges et al. (2015)

**Table 3:** Traditional career values

Career values	Definitions
Job security	Employers give/expect job security^a^
Promotion (Structured advancement)	Working way up through the ranks of a company^a, c^
Organizational focus	Career horizon is working in one organization^a, b, c^
Focus on tenure	Counting on long-term employment with one employer^a, c^
Loyalty	Reciprocal loyalty expected from organization^a, b^
Commitment	Reciprocal commitment expected from organization^a, b^
Prestige (High status)	Success is progressing up organizational ladder^a, c^

^a^Baruch (2004)

^b^Doden et al. (2018)

^c^King (2003)

The first author coded the interview data using nodes in NVivo 12.0 and an independent research assistant validated this coding. Initial agreement was approximately 90%, with all differences resolved via discussion. The other authors then checked and confirmed the coding and all authors selected representative illustrative responses.

## Results

### Protean career values

Table 4 summarizes the self-directedness and values-driven protean themes, indicating the percentage of participants making specific thematic statements and representative comments. All participants made comments that indicated that they held self-directedness attitudes, and all self-directedness themes from Table 2 were evident in the interview transcripts. The most frequent theme (79.2% of sample) was an explicit orientation toward jobs that enabled *freedom and autonomy*. Respondents preferred “the free way of doing things” (P18, M) but also indicated that they knew “where their boundaries [were]” (P16, F). Thus, they were espousing a preference for macroscopic guidelines, but did not like to be micro-managed, which was reflected in comments that they would value employers who “give me stuff and leave” (P12, F), rather than “someone standing over my shoulder and telling me what to do” (P21, M).

**Table 4:** Protean themes, percentage mentioning theme, and representative comments

Theme	%	Representative comments (participant #, gender)
*Self-directedness*		
Freedom/autonomy	79.2	I would probably prefer the free way of doing things. (P18, M)
Self-reliance	62.5	I changed my way to go independently rather than just be like my parents told me. (P11, M)
Flexibility	54.2	Having time and…flexibility to branch out for jobs or, like, projects when they come up … would be … cool. (P1, F)
Growth (Lifelong learning)	54.2	I like to be continuous, like development, like progress. (P15, M)
Networking	54.2	Having contacts or just getting to know…places of work and…people that work in them… would be…helpful (P4, F)
Self-set goals	29.2	I … have my own milestones that I'm trying to achieve. (P8, M)
Specific career plans	20.8	I want to graduate … work in the hospital for 2 years … pursue my master’s … and work overseas. (P12, F)
Proactivity	16.7	More than university … need to be approachable. … think out of the box and … help in the career. (P20, M)
Keeping updated	12.5	I keep a good plan to studying … training every day. (P23, M)
*Values driven*		
Fit to self	79.2	It’d be great to find a company that fits your values. (P17, F)
Personal satisfaction	66.7	I want to find a career that I'm happy with and makes me happy. (P7, F)
Work-life balance	62.5	I’ll have to balance other things … family … my own time … kids. (P4, F)
Suit self	50.0	Not letting other people influence my own values and what I want to do. (P6, F)
Keeping an interest	37.5	Number one, like, interests. I can imagine myself doing it and then not feeling like a job every single day. (P3, F)
Professional achievement	33.3	High qualifications … I think it’s just that strive for success. … I do want to keep moving up throughout my career to make sure that I’m constantly progressing. (P21, M)

The next most frequent theme (62.5%) was *self-reliance*. Respondents indicated that they wanted to be “in charge of the success or failure of their career” (P1, F). They expressed that career-related decision-making should be independent, with the responsibility lying with them. They did not express views that their career path was something determined by the organization that employs them. They also insisted that being financially self-sufficient was important to avoid being reliant on others.

Comments indicating that they valued *flexibility* in their jobs (54.2%) mentioned flexibility in physical space, time, and method (e.g., work at home vs. at the office, when they work, and with whom) and career direction (preference to “keep it open” [P16, F] vs. setting specific paths). They noted that acquiring “transferable” (P4, F) skills was most important as this would mean they would be employable across a range of workplaces.

*Growth (lifelong learning)* values (54.2%) were expressed in positive attitudes toward expanding their knowledge to “grow as a person” (P18, M) and taking advantage of opportunities, rather than “having a short-sighted tunnel vision of view of the world” (P15, M). They considered it negative to “be stuck in the same place and not progress” (P17, F).

For *networking* (54.2%), “referees” (P13, M), “recommendations” (P1, F), and “connections” (P1, F; P11, M; P23, M) were highlighted, and respondents reported a willingness to engage in “internships” (P19, M) and “volunteering” (P4, F) to develop useful networks before graduating.

Other protean themes, while evident, were less commonly reported. Gauging career success by *self-set goals* (29.2%) referred to them establishing their own “pathways” (P23, M) and “milestones” (P8, M) and evaluating goal achievement by their own “standards” (P17, F). *Specific career plans* (20.8%) included such things as having detailed plans for gaining qualifications and being specific about the types of workplaces that would be acceptable (e.g., self-employment, government, private sector). However, some espoused specific career plans (e.g., to “graduate by 2022 and work in the hospital for about two years and pursue my master for about a year”; P12, F). Only four participants (16.7%) valued *proactivity*, indicating, for example, that they were looking for opportunities in industry to gain extra skills and knowledge, such as “shadowing” a worker to gain insights (P5, F). Others noted it was important to be “approachable” (P20, M) to “get a resume to have an edge over other graduates” (P4, F). Three participants (12.5%) stated that it was important to *keep updated* about the labor market and potential employers in their field. They were interested in knowing what organizations were currently looking for in graduates and wanted to “keep pace with rapidly changing technologies” (P23, M) and “skills needed for the twenty-first century” (P24, M).

Being values driven was referred to by all participants except one (95.8%). They noted “my values are the most important” (P1, F) and “I would like to keep in touch with my values” (P8, M). The one exception (P13, M) indicated notable conformity to parental wishes in career decision-making and pursuit. A prevalent values-driven theme (79.2%) was a desire that their future employing organization *be a good fit* for their skills, personal qualities, and values. Many said that if a company asked something of them that would go against their values or conscience, they would “leave” (P9, F), “act” (P14, F), or “explore other job opportunities” (P12, F). They prioritized finding organizations that would allow them to be themselves and stay true to their own values; “I’ve always thought that my values would align with an organization” (P20, M).

Two-thirds of respondents indicated that *personal satisfaction* was important in defining career success. The most frequent comments included a desire for “happiness” (P6, F), “contentment” (P5, F), “being comfortable” (P15, M), “enjoyment” (P19, M), and “being satisfied” (P18, M). Many also expressed a desire to build a career that would be “meaningful and rewarding” (P18, M).

Valuing *work-life balance* (62.5%) was reflected in the insistence that the ideal balance should be “more life than work” (P24, M). They valued setting protective boundaries around their roles, relationships (e.g., family and friends), and hobbies, rather than allowing work to be the central aspect in their life. While admitting that their career was a big part of life, they believed it was important to avoid “taking work home” (P12, F).

Being driven to meet their own needs (*suit self*) was reported by 50%. They noted that it was important “not to let others drive” (P6, F) or “overly affect my career and life values” (P16, F). They wanted to rely on their beliefs and values rather than following others. One individual explained that “if someone is saying wrong for what I believe to be good, then I’m not going to just change my whole self for them” (P23, M).

*Keeping an interest* in work was mentioned by 37.5%, who saw it as important that their employment should generate “intrinsic motivation” (P21, M) and ongoing gratification (P19, M). Last, 33% referred to valuing pursuit of *professional achievement*. They referred to “achieving goals highly in my chosen fields” (P18, M), “advancing within my career” (P21, M), and by doing that, “feeling proud of fulfilling my ambitions” (P6, F).

To summarize, while all participants expressed some orientation toward career self-directedness, freedom and autonomy and self-reliance were the more commonly expressed self-directedness themes. Fewer spontaneously indicated self-directedness toward setting specific career plans, behaving proactively, and keeping updated. Of the values-driven themes, fitting work to the self, personal satisfaction, and work-life balance drove many respondents. However, keeping interested and professional achievements were not common values that drove these young adults.

### Traditional career values

Most participants (87.5%) also espoused some aspects of a traditional orientation, as summarized in Table 5. The most common traditional theme (75%) was *job security*. Participants frequently referred to a desire for this to pay bills and mortgages. They also referred to “starting a family” (P11, M) and “buying a house” (P4, F) as reasons for wanting financial security. In order to realize their lifestyle goals, they hoped to be employed in “full-time” (P10, F), rather than casual or more flexible positions. They stated that job stability could provide a comfortable, relaxed feeling, and a sense of reassurance and safety. P19 (M) referred to having already changed career direction to improve likely job security, to “be secure, and not to be worrying all the time financially.”

**Table 5:** Traditional themes, percent mentioning theme, and representative comments

Theme	%	Representative comments
Job security	75.0	Having stability … good to have a stable job. (P1, M)
Promotions (Structured advancement)	58.3	Would like to work my way up the ranks … with one company. (P16, F)
Organizational focus	41.7	I think just one. I don’t want to [be] flitting between jobs. (P12, F)
Focus on tenure	29.2	I’d probably like to stay until I resign … being at the same organization for a long time. (P14, F)
Loyalty	29.2	I’m a fairly loyal person … would prefer to be … where I can do that. (P18, M)
Commitment	8.3	You can make more of a difference…new initiatives…I like the connection. I like building relationships. (P17, F)
Prestige (High status)	4.2	Reaching a level of role or … senior position and being able to … mentor others to do what I did. (P4, F)

*Promotion (structured advancement)* within a single organization was valued by 58.3%, who wanted to “work their way up” (P16, F) or “move up” (P20, M), rather than stay stagnant in one position within their organization. They presumed that remaining in one organization would provide more opportunities, compared to moving around different organizations, to enhance their skills and obtain promotions and a higher salary.

A clear *organizational focus* (41.7%) was reflected in wishes to settle down in one organization, often for their whole careers. They did not like the idea of freelancing or frequently changing jobs. They stated that stabilizing their career in one organization was meaningful to them and that they would follow the organization’s direction and guidance because each “organization has their own paths on how to develop employees (P7, F)” and they “value organization’s rules and regulations” (P9, F). They concluded that they would feel more comfortable if they could remain there until retirement.

A *focus on tenure* (e.g., finding an ongoing job, being employed in an organization that gives stability) was mentioned by 29%, with some mentioning the value of very long-term tenure (e.g., “staying until I resign,” P14, F; staying for “30 years,” P11, M). *Loyalty* was also mentioned by 29.2% of participants. P5 (F) noted that she “wouldn’t just pack up and leave straight away.” They anticipated that demonstrating loyalty early in their careers would lead to their employers accommodating their future needs (e.g., further study, maternity leave), “being loyal is definitely going to help me out” (P3, F).

Two participants referenced *commitment*, believing that it would be possible to make more of a difference and establish new initiatives through commitment to a single organization. One stated that he would follow the employer’s directions, even if it meant compromising his own values, because “that’s part of the job” (P13, M). Last, one participant (P4, F) mentioned *prestige (high status)* by progressing up the organizational ladder, noting that “career success might be reaching a level of role or reaching senior position and being able to help or maybe mentor others to potentially do what I did.”

To summarize, the most common manner of expressing traditional values was through their desire for job security. Previous experiences and future life expectations (e.g., starting a family, purchasing a home) influenced this priority. More than half wanted the single employing organization to provide opportunities for promotions/structured advancement. However, less than one-third mentioned core traditional career values of tenure and loyalty and very few mentioned commitment and prestige.

## Discussion

Our purpose was to investigate the endorsement of, and ways of expressing, protean and traditional values by contemporary young adults using semi-structured interviews and applied thematic analysis. All participants indicated holding some protean values, confirming claims that new career orientations are present in young people (Alonderienė & Šimkevičiūtė, 2018; Creed et al., 2011; Liberato Borges et al., 2015; Rojewski et al., 2017; Sargent & Domberger, 2007). However, almost 90% also espoused some traditional career values. Thus, almost all of these young adults held a combination of protean and traditional values. The most commonly cited protean values were *freedom/autonomy* (self-directedness) and *fit to self* (values driven), which is consistent with previous literature (Abessolo et al., 2017b; Hall, 2004) and Sung et al.’s (2019) argument that young people want to self-actualize through their career. *Job security* was the dominant traditional theme, also consistent with earlier research (Gerber et al., 2009). Thus, despite most noting a desire for freedom and autonomy, they also valued the traditional benefits related to psychological work contracts (i.e., the informal “agreement between individuals and their organization” regarding reciprocation; Rousseau, 1995, p. 9). Therefore, they are likely to remain oriented to some aspects of a traditional organizational career path, at least at the early stages of their career, despite also espousing protean values. As the common desire for traditional job security also could determine future career paths and their adjustment to the workplace, our results neither support the position that traditional values are dead (Hall, 1996) nor that holding protean values is inconsistent with having a traditional orientation (Arthur et al., 1989; Hall, 2004). We found both being espoused in these young adults.

This is consistent with other empirical evidence that young adult students show co-existing orientations (Rojewski et al., 2017; Sargent & Domberger, 2007). The results align also with Sortheix et al. (2019) who found that young Europeans tended to place more importance on job security as a self-protective strategy in response to labor market disruptions, and it is possible that our results are driven, at least partly, by increasing labor market precariousness (Lewchuk, 2017), which has been exacerbated by the Covid-19 pandemic (Barua, 2020). Precarious employment is particularly prevalent among young people attempting to enter the workforce for the first time (Bessant et al., 2017), with tertiary students especially vulnerable to this (Gilfillan, 2018). Higher perceived job precariousness is related to more insecurity (Creed et al., 2020), so the commonly expressed desire for job security might be driven by this.

Once an organization begins to meet the needs and values of employees, they are likely to want to establish themselves within that structure (Wiernik & Kostal, 2019). This was reflected in the commonly espoused traditional theme of valuing structured advancement in one organization, although there was less reference to tenure, loyalty, commitment, or achieving high status within the organization. The underlying values of a career orientation might alter according to life or career developmental stage or the inherent constraints in the workplace or labor market (Hall et al., 2018). Thus, the career orientations of young adults preparing to enter the labor market might differ from those held by workers who are more established in their career. The focus on security and structure might reflect that these young adults are still studying in preparation for their career, and this need might be more associated with initial career establishment, although it could reflect differences in the current young adult population values compared to previous cohorts. For example, McDonald et al. (2005) found that Australian public sector employees valued traditional characteristics of long service and climbing the corporate ladder.

Just as not all traditional values were equally salient, neither were all protean values. Proactivity, for example, was rarely indicated spontaneously, which is counter to other evidence suggesting that there is more proactivity in seeking opportunities in the early career stage when they have relatively lower levels of personal/family commitment (Enache et al., 2011; Hall et al., 2018; Hofstetter & Rosenblatt, 2016; Kostal & Wiernik, 2017; Wiernik & Kostal, 2019). However, most of the protean themes depend on proactivity, being autonomous, relying on the self and setting ones’ own goals, valuing growth, networking, and keeping up to date all require proactivity to drive behavior, which suggests these young adults are proactive despite little explicit mentioning of it. In addition, when they enter the workforce, less commented values such as growth (lifelong learning) could be enhanced among them because employment growth in areas that require more than a bachelor’s degree is projected in the Australian labor market (National Skills Commission, 2022).

Similarly, professional achievement was not widely mentioned, which contrasts with Abessolo et al. (2017a), who found that achievement was associated positively with being values driven in a sample of Swiss workers. However, the young adults in the current study were already demonstrating the value of professional achievement by their enrollment in professional university degrees. Future research might use mixed methods to quantify the behavioral aspects of factors such as proactivity and achievement alongside qualitative data, which might give additional insight into how young people describe their important values.

Apart from the likely influences of the perceived current and predicted future labor market, there are other reasons why young people might hold a mix of protean and traditional career values. First, Baruch (2014) noted that career choice might be associated with these orientations. Most students in our sample were completing a professional degree (e.g., physiotherapy, psychology, law), which leads directly to traditional professions involving prescribed career paths where experience is often gained initially in organizations, such as hospitals, clinics, and large firms, before entering more independent arrangements, such as self-employment and contract work. In Australia, many professional graduates have mandatory supervised practice requirements to meet before they can be fully registered as professionals (www.ahpra.gov.au). These requirements are often met best in large organizations with well-developed training and development structures, which might help explain the mix of a desire for security and structure with less interest in long-term tenure alongside interest in freedom and autonomy and proactivity being less salient. Thus, young students who chose traditional professions might more strongly endorse traditional career values than students with more generic degrees, like liberal arts. For students preparing for less well-defined and less traditional careers, proactivity (e.g., via entrepreneurship), and professional achievement might be more salient due to a higher need to forge more independent career paths from the beginning (e.g., the Silicon Valley model in the USA, where entrepreneurship is valued; Audretsch, 2021).

This expression of a mixture of values is consistent with an individualism perspective (Takeuchi et al., 2021), whereby individuals adopt values that are functional for the situation and have these values reinforced by their expression and application. For example, young adult professionals would come to understand the need for post-graduate supervision and have internalized the need for finding work where they can find this (e.g., in an organization), and have this acknowledgment reinforced while at university. At the same time, they would expect and have reinforced the need for independence and autonomy to apply their knowledge and skills. Schwartz et al. (2012) stressed that healthy value systems need to be coherent, self-protective, generate compatible drives, and stimulate personal growth. In our example, students’ holding values related to both organizational commitment and personal independence is consistence with Schwartz’s propositions.

### Implications

The findings have theoretical and practical implications. They contribute to a better understanding of new career theories (Hall et al., 2018) by illustrating that young people might express a mixture of protean and traditional career values. The narratives revealed that freedom/autonomy, a central self-directedness component of the protean career, was the most widespread reported value for these young adults, and they also expressed a range of other protean values, such as self-reliance, preference for work-life balance, and need for personal satisfaction. Equally common, however, were the preferences for job security and structured advancement—largely traditional career values—which have been neglected in new career theories and conceptualizations of young adult career approaches. Our results indicated that aspects of traditional orientation should be included as career values for young adults in order to yield a more precise and developmentally appropriate young adult career theory.

In practice, recruiters and human resource managers need to be aware that, while young adults might expect freedom, autonomy, and fit with their own values in an ideal workplace, they will still, at least initially, seek job security and structured opportunities for advancement. This might not translate into long-term tenure in all cases, although almost half of our participants did refer to preferring ongoing employment in one organization, consistent with a traditional career path. This means, for example, that when recruiting young people, organizations should provide realistic job descriptions, which can be reinforced in job interviews and orientation programs, so that young people can make an informed choice about the work and conditions being offered and whether they are a good fit for them. This will benefit both the young person, who can select employment that meets their needs and suit organizations as they avoid selecting staff that have expectations that clash with their goals. The lesson for young people is to be clear about their values, do their homework on what employers are offering, and seek to match their most salient values with the conditions on offer.

Additionally, tertiary education institutions need to educate students about twenty-first century career contexts as they are likely to be different from those of previous generations (e.g., need for greater career mobility; Alonderienė & Šimkevičiūtė, 2018; Sortheix et al., 2019) and that placing too heavy a reliance on security and structured advancement within one organization could be unrealistic and potentially harmful. Indeed, Sortheix et al. (2015) found that a focus on security values at age 21 were related to a higher possibility of unemployment two years later. Providing work experience and networking opportunities could help develop more realistic views of potential career paths and might allow young people test and shape their values against the requirements of the labor market. Universities also could provide or strengthen their structured avenues for values and career exploration (e.g., values and career workshops, online courses, careers counselors) and embed career-related material in their courses and programs so that by the end of their formal study students have realistic expectations of the type of work, and requirements for that work that will suit them.

### Limitations and conclusion

Our study contributes to a better understanding of young adults’ career values. However, our sample was limited to young professionals in training (largely majoring in social sciences) at one university in Australia, which is a developed, individualistic country; thus, our sample was not representative of young people generally, and our findings might not generalize to the broader young adult population. Some studies have shown that that career values vary across different cultural settings (Aygün et al., 2008; Wong & Yuen, 2012). Thus, future research should examine career values in other samples of young adults, including those with different cultural backgrounds (e.g., collectivist students), demographic factors (e.g., SES), educational background (e.g., those in non-tertiary training, such as in vocational colleges; those who left school early to enter the workforce directly), and work experience (e.g., number of jobs held, accumulated work experience). Also, young people still completing their education have limited work experience in their chosen career, so it remains unclear whether our results reflect stable orientations developed largely through the influence of, and experiences within, their families of origin, community, and education, or reflect ideals that might change considerably post-graduation as they gain experiences in their chosen career and the adult labor market. Graduates need to adjust as they encounter incongruencies between ideal expectations and actual experiences in their early career period (Riordan & Goodman, 2007), which potentially will alter their attitudes and moderate their values. Studies that track young people over time should add to our understanding of how and when these values might change.

In conclusion, we demonstrated that protean and traditional values co-existed in young adults who were completing their education and before they experienced their first career jobs. Not all protean and traditional values were espoused, with only a small number of each being salient to most of our sample. Thus, the traditional career might not be dead as claimed (Hall, 1996), and the full range of protean values might not be held strongly by young people as claimed by new career theorists. By retaining the most relevant traditional career values alongside the more salient protean values in a comprehensive theory of career values, young people’s expectations and behaviors when entering the world of work are likely to be more predictable.

## Appendix

### Interview protocol: the underlying career values of young people’s protean and traditional career orientations

- Welcoming comments, “thank you” statement for participating, boundary setting (e.g., voluntary and anonymous nature of interview), and anticipated path of interview.
- Broad introductory questions: “Can you explain to me…”
  - How and why you chose your degree?
  - What were your career goals?
- More targeted questions regarding who and what were important in your career decisions, progress, and success:
  - What/who have you considered when choosing your career?
  - What effort have you made for your future career?
  - How do you think such efforts will influence your future career?
  - What do you think are the most important factors for your future career progress?
  - How do you view your career prospects in terms of your future employability?
  - How would you define career success and what would that mean to you?

Some representative illustrative questions

- What aspects of your career selection appealed to you?
- Can you paint a picture of what it would be like to work your career in the one company?
- What would it be like to work in your own business/start your own private practice?
- What sort of career would you have if you completed a higher degree?
- What was it about university that drew you to it?
- What was it about your discipline that attracted you?
- What would be the deciding factors when selecting somewhere to work after you completed your education?
- What would motivate you to remain/leave a work situation?
- What values do you hold that will influence where you work?
  - What values would you not want to compromise on?
  - What aspects of your current/past (part-time, casual) work have you enjoyed?
  - Is there something that you think you would value in a future career?
